# Supplementary material for: Components of the full blood count as risk factors for colorectal cancer detection: a systematic review protocol
Source: BMJ Open. 2019 Dec 16;9(12):e032759. doi: 10.1136/bmjopen-2019-032759 (PMC6937079; doi:10.1136/bmjopen-2019-032759)
Supplement: Supplementary data [file bmjopen-2019-032759supp001.pdf]

## Proposed MEDLINE (OVID) search strategy

Database and Platform to be searched: Medline (Ovid MEDLINE® Epub Ahead of Print, In-Process & Other Non-Indexed Citations, Ovid MEDLINE® Daily and Ovid MEDLINE®) 1946 to present.

1. Colonic Neoplasms/bl [Blood]
2. Colonic Neoplasms/di [Diagnosis]
3. Colonic Neoplasms/ep [Epidemiology]
4. Colorectal Neoplasms/di [Diagnosis]
5. Colorectal Neoplasms/ep [Epidemiology]
6. Colorectal Neoplasms/bl [Blood]
7. Rectal Neoplasms/bl [Blood]
8. Rectal Neoplasms/di [Diagnosis]
9. Rectal Neoplasms/ep [Epidemiology]
10. Adenomatous Polyposis Coli/
11. Sigmoid Neoplasms/
12. Colorectal Neoplasms, Hereditary Nonpolyposis/
13. ((colorectal or bowel or colon or colonic or rectal or rectum) adj3 (cancer$ or carcinoma$ or adenoma$ or neoplas$ or metasta$ or carcinogen$ or tumour$ or tumor$ or malignan$)).ti,ab,kw.
14. or/1-13
15. exp Blood Cell Count/
16. exp Hemoglobins/
17. Blood Platelets/
18. Neutrophils/
19. Basophils/
20. Eosinophils/
21. Lymphocytes/
22. Monocytes/
23. Occult Blood/
24. Thrombocytosis/
25. Leukocytosis/

26. Lymphocytosis/

27. Eosinophilia/

28. Anemia/

29. Leukopenia/

30. Neutropenia/

31. Lymphopenia/

32. Thrombocytopenia/

33. Polycythemia/

34. Erythrocytes/

35. Leukocytes/

36. Pancytopenia/

37. ((blood or platelet) adj2 count$).ti,ab,kw.

38. (CBC or FBC).ti,ab,kw.

39. (blood adj2 exam$).ti,ab,kw.

40. (haematolog$ or hematolog$ or haemoglobin or hemoglobin or haematocrit or hematocrit).ti,ab,kw.

41. ((red or white) adj1 blood adj1 cell$).ti,ab,kw.

42. (mean adj1 (platelet or corpuscular) adj1 volume$).ti,ab,kw.

43. (mean adj1 corpuscular adj1 (haemoglobin or hemoglobin)).ti,ab,kw.

44. (platelet$ or basophil or basophils or eosinophil or eosinophils or lymphocyte$ or monocyte$ or neutrophil or neutrophils or erythrocyte$ or leukocyte$).ti,ab,kw.

45. (blood adj1 (test$ or draw$)).ti,ab,kw.

46. (neutrophilia or monocytosis or basophilia or anemia or anaemia or monocytopenia or eosinopenia or basopenia or thrombocytopenia or leucocytosis or lymphocytosis or eosinophilia or leucopenia or neutropenia or lymphopenia or pancytopenia or polycythemia or bicytopenia).ti,ab,kw.

47. or/15-46

48. (abnormalit$ or diagnos$ or "pre-diagnos$" or prediagnos$ or change$ or detect$ or elevat$ or distribut$ or deficien$ or identif$ or presence or indicati$ or determin$ or undiagnosed or definition$ or alteration$).ti,ab,kw.

49. (predict$ or prognos$).ti,ab,kw.

50. (risk adj1 (predict$ or marker$ or scor$)).ti,ab,kw.

51. Predictive Value of Tests/

52. Probability/
53. Prognosis/
54. Risk Factors/
55. Risk Assessment/
56. Incidence/
57. or/48-56
58. 14 and 47 and 57
