# Supplementary material for: Components of the full blood count as risk factors for colorectal cancer detection: a systematic review protocol
Source: BMJ Open. 2019 Dec 16;9(12):e032759. doi: 10.1136/bmjopen-2019-032759 (PMC6937079; doi:10.1136/bmjopen-2019-032759)
Supplement: Author's manuscript [file bmjopen-2019-032759.draft_revisions.pdf]

BMJ Open is committed to open peer review. As part of this commitment we make the peer review history of every article we publish publicly available.

When an article is published we post the peer reviewers' comments and the authors' responses online. We also post the versions of the paper that were used during peer review. These are the versions that the peer review comments apply to.

The versions of the paper that follow are the versions that were submitted during the peer review process. They are not the versions of record or the final published versions. They should not be cited or distributed as the published version of this manuscript.

BMJ Open is an open access journal and the full, final, typeset and author-corrected version of record of the manuscript is available on our site with no access controls, subscription charges or pay-per-view fees (http://bmjopen.bmj.com).

If you have any questions on BMJ Open's open peer review process please email info.bmjopen@bmj.com

BMJ Open

Components of the full blood count as risk factors for colorectal cancer detection: a systematic review protocol

Journal:	*BMJ Open*
Manuscript ID	bmjopen-2019-032759
Article Type:	Protocol
Date Submitted by the Author:	03-Jul-2019

Complete List of Authors:	Virdee, Pradeep; University of Oxford, Centre for Statistics in Medicine<br>Kirtley, Shona; University of Oxford, Centre for Statistics in Medicine<br>Elhussein, Leena; University of Oxford, Centre for Statistics in Medicine<br>Watkinson, Peter; Oxford University Hospitals NHS Trust, Kadoorie Centre for Critical Care research and Education<br>Holt, Tim; Oxford University, Department of Primary Care Health Sciences<br>Birks, Jacqueline; University of Oxford, Centre for Statistics in Medicine

Keywords:	Colorectal cancer, Full blood count, Complete blood count, Early detection, Risk factor

SCHOLARONE™
Manuscripts

Title

Components of the full blood count as risk factors for colorectal cancer detection: a systematic review protocol

Authors and affiliations

Pradeep S. Virdee[1], Shona Kirtley[1], Leena Elhussein[1], Peter Watkinson[2], Tim Holt[3], Jacqueline Birks[1]

[1]Centre for Statistics in Medicine, NDORMS, University of Oxford, UK

[2]Nuffield Department of Clinical Neurosciences, John Radcliffe Hospital, Oxford, UK

[3]Nuffield Department of Primary Care Health Sciences, University of Oxford, University of Oxford, UK

Pradeep S. Virdee (PSV): pradeep.virdee@csm.ox.ac.uk

Shona Kirtley (SK): shona.kirtley@csm.ox.ac.uk

Leena Elhussein (LE): leena.elhussein@csm.ox.ac.uk

Peter Watkinson (PW): peter.watkinson@ndcn.ox.ac.uk

Tim Holt (TH): tim.holt@phc.ox.ac.uk

Jacqueline Birks (JB): jacqueline.birks@csm.ox.ac.uk

Corresponding author

Author: Pradeep S. Virdee

Address: CSM Botnar, Nuffield Orthopaedic Centre, Windmill Road, University of Oxford, OX3 7LD, UK

Email: pradeep.virdee@csm.ox.ac.uk

Telephone: 01865737950

For peer review only

Abstract

Introduction

Colorectal cancer is the fourth most common type of cancer and the second most common cause of cancer-related death in the UK. The full blood count (FBC), a commonly used blood test, may play a role in early detection of the disease. Previous studies have aimed to identify how levels of individual components, such as haemoglobin, can be used to assist diagnosis. We aim to systematically review studies to identify whether components of the FBC are risk factors for diagnosis of colorectal cancer, critically appraise the methods used to assess the association, and, where reported, assess how well the FBC components perform..

Methods and analysis

The MEDLINE (via OVID), EMBASE (via OVID), CINAHL (via EBSCOhost), and Web of Science databases will be searched to identify in-human studies reporting the association between levels of at least one component of the FBC and risk of a future diagnosis of colorectal cancer. Clincialtrials.gov and the World Health Organisation (WHO) registry will be searched to identify relevant on-going research. Search terms will include relevant Medical Subject Headings (MeSH) and Emtree headings and free-text terms relating to the blood count, colorectal cancer, and diagnosis. No date or language restrictions will be applied. Two reviewers will independently identify studies for inclusion and perform data extraction. Time intervals between the blood tests and diagnosis will form subgroups for analysis.

Ethics and dissemination

There is no direct patient involvement and only published articles will be systematically reviewed; no ethical approval is required. Results from this review will set a foundation for intended future work on developing a new risk score for early detection of colorectal cancer, derived using FBC data. This systematic review will also provide guidance on the analysis of time to diagnosis.

For peer review only - http://bmjopen.bmj.com/site/about/guidelines.xhtml

Systematic review registration

International prospective register of systematic reviews (PROSPERO: CRD42019134400)

Strengths and limitations of this study

- The first systematic review to identify the role of components of the full blood count from a blood test in detection of colorectal cancer.
- As the number of studies reporting on the association between components of the full blood count and diagnosis of colorectal cancer is increasing over time, this review is timely.
- This systematic review will make recommendations for the development of intendend future risk scores for early detection of colorectal cancer, derived using full blood count data.
- This review will be limited to examining published articles, so the use of blood count values in diagnosis of colorectal cancer as per local practices' policy will not be included, unless published.

Introduction

Colorectal cancer is the fourth most common type of cancer in the UK, with around 41,800 new cases diagnosed in 2015[1]. It is the second most common cause of cancer-related death in the UK, with around 16,400 deaths in 2016[2]. Colorectal cancer develops slowly from pre-cancerous polyps that may be present for years before becoming malignant. It often goes unnoticed until patients start showing symptoms, such as abdominal pain, weight loss, and change in bowel habits. At this point, the cancer has usually developed to a stage where it is difficult to treat and cannot be surgically removed[3]. The stage at diagnosis heavily influences survival. Five-year survival is 95% if the cancer is diagnosed at Stage I, where the cancer is confined to the bowel lining, but 7% if at Stage IV, where the cancer has spread to other organs[4].

For peer review only

Patients with colorectal cancer respond well to existing interventions, such as surgery, chemotherapy, and radiotherapy, if the cancer is diagnosed at an early stage. Currently, over 50% of patients are diagnosed with late-stage cancer (Stage III and IV), with approximately half of these having metastases at diagnosis, compared with the earlier stages (Stage I and II). Their outcomes are much poorer than those diagnosed with cancer in an earlier stage, including survival[5]. Symptom-based approaches to identify risk of colorectal cancer exist, such as QCancer Colorectal, a statistical prediction model widely used in UK primary care practices[6]. However, approaches to detect cancer earlier, before overt symptoms appear, would be of considerable benefit. Early detection and removal of polyps can prevent colorectal cancer from developing and improve survival.

A full blood count (FBC) is a common blood test ordered by a doctor in both primary and secondary care, because abnormalities could relate to a wide range of diseases and conditions used in clinical practices. A FBC incudes up to 20 blood components (red blood cells, white blood cells, mean platelet volume, haemoglobin, haematocrit, mean corpuscular volume, mean corpuscular haemoglobin, mean corpuscular haemoglobin concentration, red blood cell distribution width, platelet, basophil #, basophil %, eosinophil #, eosinophil %, lymphocyte #, lymphocyte %, monocyte #, monocyte %, neutrophil #, neutrophil %).

It is known that the FBC may play a role in the diagnosis of colorectal cancer, with subtle changes in the FBC developing when the cancer is at a relatively early stage. Some studies have explored the relationship between levels of specific components of the FBC and colorectal cancer diagnosis[7, 8]. For example, anaemia in FBC data from UK primary care predicts risk of colorectal cancer and iron deficiency is an independent risk factor[8]. In the last few years, a number of individual studies have reported on the association between components of the FBC, including haemoglobin, platelet count, and red cell distribution width, and diagnosis of colorectal cancer[9-13]. Furthermore, risk scores have

For peer review only

For peer review only - http://bmjopen.bmj.com/site/about/guidelines.xhtml

recently been developed using methods that incorporate FBC data as predictors for colorectal cancer diagnosis. This includes the statistical prediction model QCancer Colorectal[6], which includes haemoglobin level as a predictor for risk of diagnosis, and other algorithms, such as the ColonFlag, an Israeli risk score derived recently from FBCs using machine-learning methods[14].

The aim of this review is to identify components of the FBC as potential risk factors for colorectal cancer diagnosis, given the increasing interest in the use of a FBC for early detection, and to inform the development and validation of future risk scores for early detection of colorectal cancer.

Existing systematic reviews

Systematic reviews of colorectal cancer detection already exist, four of which have been identified as reviews of blood-based characteristics. However, none of these reviews focused on the FBC blood test.

Bhardwaj *et al*[15] and Shah *et al*[16] focused on reviewing parts of the blood, such as blood-based proteins, DNA biomarkers, and gene expressions, that are not part of a FBC. Nikolou *et al*[17] reviewed blood markers that were divided into four groups: nucleic acids, cytokines, antibodies, and proteins, which are different components of the blood to the FBC. Del Giudice *et al*[18] reviewed clinical features of suspected characteristics, but this consisted primarily of symptoms reported in clinic. Their findings include the predictive performance of haemoglobin, but they do not report on the remaining blood components that form the FBC, including up to 19 other components, which are also our main interest here. Usher-Smith *et al*[19] performed a comprehensive appraisal of prediction models for risk of colorectal cancer. However, these were not necessarily models containing components of the FBC as risk factors. Additionally, other types of studies were excluded from their review, such as retrospective cohort and case-control studies, which explored the association between components of the FBC and risk of colorectal cancer diagnosis.

For peer review only - http://bmjopen.bmj.com/site/about/guidelines.xhtml
For peer review only

Other reviews for colorectal cancer detection have a different focus to the review described here. These include Astin *et al*[20], who reviewed the diagnostic value of symptoms, Williams *et al*[21], focusing solely on prediction models for patients who reported symptoms, and Vega *et al*[22], who summarised the available evidence concerning the diagnostic process, its pitfalls, and opportunities.

To our knowledge, there are no existing reviews identifying components of the FBC as risk factors for colorectal cancer diagnosis.

Research aims

In this review, we aim to identify and appraise studies that explored the association between levels of blood components from a FBC and risk of diagnosis of colorectal cancer. We aim to:

1) Identify which specific blood component(s) was investigated and whether it was considered associated with risk of diagnosis of colorectal cancer.
2) Describe the study design and methods used to assess the association. For example, whether statistical methods were used, whether blood levels were modelled as categorical or continuous, how missing data were dealt with, and the timeframe to diagnosis.
3) Describe the study population and setting. For example, whether the study used electronic health data, primary or secondary care patients, and symptomatic or asymptomatic patients.
4) Identify whether the values of the components of a FBC differ between those diagnosed with colorectal cancer and those not diagnosed.

Studies reporting the development, validation or testing of a risk score that uses FBC component(s) for colorectal cancer diagnosis, such as a prediction model, will also be included. For these studies, the following information will be collected, in addition to the points above:

For peer review only - http://bmjopen.bmj.com/site/about/guidelines.xhtml

5) Report the weight the blood component(s) has on risk of diagnosis of colorectal cancer. For example, this could be the coefficient from a regression model.
6) Identify risk factors, in addition to the components of a FBC, used to derive risk scores.
7) Report which risk scores have undergone validation, whether it was internal and/or external, and how well the risk scores performed.

Methods

This systematic review protocol follows the Preferred Reporting Items for Systematic review and Meta-Analysis Protocols (PRISMA-P) guidelines[23]. This review protocol was registered with the International Prospective Register of Systematic Reviews database on 13th May 2019 (PROSPERO: CRD42019134400). Screening of studies is expected to commence in August 2019. The review will be reported in accordance with the Preferred Reporting Items for Systematic Reviews and Meta-Analyses (PRISMA) checklist[24].

Participants

All adult (aged 18 years or above) populations will be considered, with the exception of those who are studied post-diagnosis of colorectal cancer.

Search strategy

The MEDLINE (via OVID), EMBASE (via OVID), CINAHL (via EBSCOhost), and Web of Science databases will be searched to identify publications in the medical literature that report the association between components of the FBC and diagnosis of colorectal cancer. Additionally, clincialtrials.gov and the World Health Organisation (WHO) registry will be searched to identify any relevant on-going research. Search terms will include relevant Medical Subject Headings (MeSH) and EMTREE headings, and free-text search terms, which will be searched for in the title, abstract, or keyword fields. Free-text search terms will include synonyms and related variants of the blood count, such as “platelet” or “basophil”,

For peer review only - http://bmjopen.bmj.com/site/about/guidelines.xhtml

of colorectal cancer-related terms, such as “bowel” or “rectal”, and of detection, such as “diagnosis” or “prediction” related terms. There will be no date or language restrictions applied to the search. The proposed full search strategy for the MEDLINE database can be found in Supplementary file 1.

Study selection

Data management

The results from each of the database searches will be downloaded into Rayyan[25], a web-tool for systematic reviews, and the full results set de-duplicated and screened. Screening of each publication will be performed independently by two reviewers. Each reviewer will read titles and abstracts to identify the study sample for analysis, using pre-specified selection criteria. Disagreements between the two reviewers will be discussed until agreement is reached. In the event that no agreement is reached, a third reviewer will be consulted for adjudication. The search results and study selection process will be reported using a PRISMA flow diagram[24].

Selection criteria

We will include any in-human primary research publication reporting the association between the value of at least one of the individual components of a FBC and the risk of a future diagnosis of colorectal cancer. For studies reporting the development and/or validation of a risk score, these will only be included if the risk score was derived using at least two risk factors, with at least one being a component of the FBC.

We will exclude abstracts and conference proceedings, as they are likely to produce incomplete data for a thorough review. As the main interest is in the recorded value of a blood component from a FBC, studies not investigating the value itself will be excluded, such as studies exploring the morphology of individual blood components. Studies using a cross-sectional design will be excluded as the data reflects a ‘snapshot’ at a certain time, hence cannot predict future risk. Clinical trials will be excluded

For peer review only

For peer review only - http://bmjopen.bmj.com/site/about/guidelines.xhtml

as our interest is in FBC data, not intervention. Existing systematic reviews, correspondence, and case studies pertaining to single individuals will be excluded.

Data extraction

Data management

Data extracted from the publications included for analysis is intended to be recorded in the Research Electronic Data Capture (REDCap) electronic tool[26]. Two reviewers will read full-text publications and independently extract data from each study. Disagreements between the two reviewers will be resolved by a third reviewer.

Data items

Data extracted will include items from the PRISMA checklist[24]. Data specific to this study will be extracted using a self-developed data extraction form. This form will be piloted on a small number of publications in the final sample; the publications will be selected randomly and the number chosen for piloting will depend on number of publications included in the sample for analysis. The study aims specify the data items for extraction, which will include the following:

- Study characteristics (such as authors, year of publication, sample size)
- Study design (such as prospective, cohort, case-control)
- Study setting (such as geographical location, primary or secondary care, electronic health record data)
- Population characteristics (such as age, gender, symptomatic/asymptomatic)
- Outcome details (such as number of cases/controls, timeframe)
- Blood component details (such as which component was analysed, blood levels used, its association with risk of colorectal cancer)
- Methods applied to assess the association (such as how missing data were handled, whether blood levels were categorised, statistical methods used)

For peer review only

For peer review only - http://bmjopen.bmj.com/site/about/guidelines.xhtml

- Differences between those with and without a diagnosis of colorectal cancer, where possible (such as whether blood levels differ between the two groups).

For the subset of studies reporting the development, validation or testing of a risk score for colorectal cancer diagnosis that incorporates FBC data, the following data items will also be collected:

- Weight of blood component(s) on risk (such as model coefficient, 95% confidence interval)
- Risk factors used in addition to the blood component(s) (such as age, gender)
- Measures of discrimination and calibration (such as sensitivity, specificity)

Data analysis and synthesis

Missing data

We will contact the publication's authors to request missing or incomplete data. If the data are not obtained, we will provide a description of the missing data for each study.

Analysis methods

Quantitative data will be summarised using descriptive statistics, such as means with standard deviations or medians with inter-quartile range for continuous data and counts with proportions for categorical data, and narrative synthesis. Summaries will also be provided in graphical and tabular form.

Where appropriate and feasible, we will use random-effects meta-analysis and forest plots to pool data across studies, with the I^2 statistic to assess heterogeneity. The application of statistical methods for analysis will be informed by the number of publications with common measures. For example, if many case-control studies report effect estimates, such as odds ratios or risk ratios of blood component levels between those with and without a diagnosis of colorectal cancer, effect estimates

For peer review only

For peer review only - http://bmjopen.bmj.com/site/about/guidelines.xhtml

will be pooled using a random-effects meta-analysis. For all statistical analyses, a two-sided 5% significance level will be used.

Subgroups for analysis

The time-period between the blood test and diagnosis of colorectal cancer will be collected, such as whether the studies report two-year or five-year risk of diagnosis. It is intended that time intervals will be separated into bands, such as 6-monthly or yearly intervals before diagnosis, forming subgroups for analysis. Analyses will be performed for each time band separately. These results will set a foundation for intended future work on the analyses of time to diagnosis.

Planned future work will use findings from this review to develop a new risk score for early detection of colorectal cancer in the UK. Hence, studies performed in the UK population will form a subgroup for analysis.

Assessment of bias

Risk of bias, such as analysis bias and publication bias, in each study will be assessed using tools appropriate to the design of the study. For example, the Quality In Prognosis Studies (QUIPS) tool[27] may be used for studies of associations and the Cochrane Prediction model Risk Of Bias ASsessment Tool (PROBAST)[28] may be used for the subset identified as prediction modelling studies. Studies considered to have a high risk of bias will be excluded in a sensitivity analysis.

Ethics and dissemination

Ethical approval is not required for this systematic review as there will be no direct patient investigations in this study and only published articles will be systematically reviewed. Results from this systematic review will be published in an open access journal.

For peer review only

For peer review only - http://bmjopen.bmj.com/site/about/guidelines.xhtml

Patient and public involvement

Patients and members of the public reviewed this protocol and will review the results to apply a patient perspective to the research.

Discussion

Many studies have explored the role that individual components of a FBC may have in the diagnosis of colorectal cancer. Such exploration has recently become popular. However, no changes have been incorporated widely in general practice to make use of such data for early detection of the disease. Despite existing methods for colorectal cancer detection, such as screening, and methods to assist detection, such as the widely-used prediction model QCancer Colorectal, the lack of early detection remains a major health problem, with the majority of colorectal cancers in the UK diagnosed at a late stage[5]. The ability to identify cases of colorectal cancer at a time point earlier than existing methods would be of considerable benefit and could save many lives.

Our team has available a set of FBC data from a large cohort of patients from UK primary care practices. However, optimal strategies for modelling the data, such as handling biologically implausible blood values and analysing changes over time, are unclear. This review will provide guidance on the specific components to analyse and statistical modelling strategies to implement. Intended future work includes developing and validating a new risk score for early detection of colorectal cancer, derived using statistical modelling of FBC components.

Funding

This study is funded by the National Institute for Health Research (NIHR) Doctoral Research Fellowship programme (DRF-2018-11-ST2-057). The funders had no role in the development of this protocol.

For peer review only

For peer review only - http://bmjopen.bmj.com/site/about/guidelines.xhtml

Competing interests

The authors declare that they have no competing interests.

Authors' contributions

PSV, TH, and JB developed the study and research question. PSV developed this systematic review protocol. SK developed the search strategy, with input from PSV and LE and oversight from PW, TH, and JB. PSV developed the data extraction sheet, with input from LE and oversight from PW, TH, and JB. PSV and SK will perform the final study search. PSV and LE will perform screening, data extraction, and analysis under the supervision of PW, TH, and JB. PSV drafted this protocol and all authors developed and approved the final manuscript before submission.

Acknowledgements

The authors would like to thank Pete Wheatstone, Margaret Ogden, and Julian Ashton for their input into the protocol as patient and public involvement (PPI) representatives.

References

[1] Cancer Research UK. Bowel Cancer Incidence. https://www.cancerresearchuk.org/health-professional/cancer-statistics/statistics-by-cancer-type/bowel-cancer/incidence#heading-Two (last accessed 3 May 2019).

[2] Cancer Research UK. Bowel Cancer Mortality. https://www.cancerresearchuk.org/health-professional/cancer-statistics/statistics-by-cancer-type/bowel-cancer/mortality (last accessed 3 May 2019).

For peer review only - http://bmjopen.bmj.com/site/about/guidelines.xhtml

[3] Winawer S.J., Zauber A.G., Ho M.N., O'Brien M.J., *et al* (1993). Prevention of Colorectal Cancer by Colonoscopic Polypectomy. *N. Engl. J. Med*. 329:1977–1981.

[4] Cancer Research UK. Bowel Cancer Survival Statistics. Available at https://www.cancerresearchuk.org/health-professional/cancer-statistics/statistics-by-cancer-type/bowel-cancer/survival#heading-Three (last accessed 3 May 2019).

[5] Cancer Research UK. Bowel Cancer Incidence. Available at http://www.cancerresearchuk.org/health-professional/cancer-statistics/statistics-by-cancer-type/bowel-cancer/incidence#heading-Three (last accessed 3 May 2019).

[6] Hippisley-Cox J., Coupland C. (2015). Development and validation of risk prediction algorithms to estimate future risk of common cancers in men and women: prospective cohort study. *BMJ Open.* 5:e007825.

[7] Spell D.W., Jones D.V. Jr, Harper W.F., Bessman D.J., *et al* (2004). The value of a complete blood count in predicting cancer of the colon. *Cancer Detection and Prevention*. 28:37–42.

[8] Hamilton W., Lancashire R., Sharp D., Peters T.J., *et al* (2008). The importance of anaemia in diagnosing colorectal cancer: a case-control study using electronic primary care records. *Br. J. Cancer*. 98:323–327.

[9] Bailey SER., Ukoumunne O.C., Shephard E.A., Hamilton W., *et al* (2017). Clinical relevance of thrombocytosis in primary care: a prospective cohort study of cancer incidence using English electronic medical records and cancer registry data. *Br. J. of Gen Pract*. 67(662):e405-e413.

For peer review only

For peer review only - http://bmjopen.bmj.com/site/about/guidelines.xhtml

[10] Goshen R., Mizrahi B., Akiva P., Kinar Y., *et al* (2017). Predicting the presence of colon cancer in members of a health maintenance organisation by evaluating analytes from standard laboratory records. *British Journal of Cancer*. 116:944–950.

[11] Pilling L.C., Atkins J.L., Kuchel G.A., Ferrucci L., *et al* (2018). Red cell distribution width and common disease onsets in 240,477 healthy volunteers followed for up to 9 years. *PLOS ONE*. 13(9):e0203504.

[12] Song Y., Huang Z., Kang Y., Lin Z., *et al* (2018). Clinical Usefulness and Prognostic Value of Red Cell Distribution Width in Colorectal Cancer. *BioMed Research International*. 9858943:1-7.

[13] Ankus E., Price S.J., Ukoumunne O.C., Hamilton W., *et al* (2018). Cancer incidence in patients with a high normal platelet count: a cohort study using primary care data. *Family Practice*. 35(6):671–675.

[14] Kinar Y., Kalkstein N., Akiva P., Levin B., *et al* (2016). Development and validation of a predictive model for detection of colorectal cancer in primary care by analysis of complete blood counts: a binational retrospective study. *J Am Med Inform Assoc*. 23:879–890.

[15] Bhardwaj M., Gies A., Werner S., Schrotz-King P., *et al* (2017). Blood-Based Protein Signatures for Early Detection of Colorectal Cancer: A Systematic Review. *Clinical and Translational Gastroenterology*. 8:e128.

[16] Shah R., Jones E., Vidart V., Kuppen P.J., *et al* (2014). Biomarkers for Early Detection of Colorectal Cancer and Polyps: Systematic Review. *Cancer Epidemiol Biomarkers Prev*. 23(9):1712-1728.

For peer review only

For peer review only - http://bmjopen.bmj.com/site/about/guidelines.xhtml

[17] Nikolaou S., Qiu S., Fiorentino F., Rasheed S., *et al* (2018). Systematic review of blood diagnostic markers in colorectal cancer. *Techniques in Coloproctology*. 22:481–498.

[18] Del Giudice M.E., Vella E.T., Hey A., Simunovic M., *et al* (2014). Systematic review of clinical features of suspected colorectal cancer in primary care. *Canadian Family Physician*. 60:e405-e415.

[19] Usher-Smith J.A., Walter F.M., Emery J.D., Win A.K., *et al* (2016). Risk Prediction Models for Colorectal Cancer: A Systematic Review. *Cancer Prev Res*. 9(1):13–26.

[20] Astin M., Griffin T., Neal R.D., Rose P., *et al* (2011). The diagnostic value of symptoms for colorectal cancer in primary care: a systematic review. *Br. J. of Gen Pract*. 61(586):e231-e243.

[21] Williams T.G.S., Cubiella J., Griffin S.J., Walter F.M., *et al* (2016). Risk prediction models for colorectal cancer in people with symptoms: a systematic review. *BMC Gastroenterology*. 16(63):1-16.

[22] Vega P., Valentín F., Cubiella J. (2015). Colorectal cancer diagnosis: pitfalls and opportunities. *World J Gastrointest Oncol*. 7(12):422-433.

[23] Moher D., Shamseer L., Clarke M., Ghersi D., *et al* (2015). Preferred Reporting Items for Systematic Review and Meta-Analysis Protocols (PRISMA-P) 2015 statement. *Systematic Reviews*. 4(1):1.

[24] Moher D., Liberati A., Tetzlaff J., Altman D.G., *et al* (2009). Preferred Reporting Items for Systematic Reviews and Meta-Analyses: The PRISMA Statement. *PLoS Med*. 6(7):e1000097.

[25] Mourad O., Hammady H., Fedorowicz Z., Elmagarmid A. (2016). Rayyan — a web and mobile app for systematic reviews. *Systematic Reviews*. 5:210.

For peer review only

For peer review only - http://bmjopen.bmj.com/site/about/guidelines.xhtml

[26] Harris P.A., Taylor R., Thielke R., Payne J., *et al* (2009). Research electronic data capture (REDCap) – A metadata-driven methodology and workflow process for providing translational research informatics support. *J Biomed Inform*. 42(2):377-81.

[27] Hayden J.A., van der Windt D.A., Cartwright J.L., Cote P.C. (2013). Assessing Bias in Studies of Prognostic Factors. *Annals of Internal Medicine*. 158:280-286.

[28] Wolff R.F., Moons K.G.M., Riley R.D., Whiting P.F., *et al* (2019). PROBAST: A Tool to Assess the Risk of Bias and Applicability of Prediction Model Studies. *Annals of Internal Medicine*. 170:51-58.

For peer review only

For peer review only - http://bmjopen.bmj.com/site/about/guidelines.xhtml

Proposed MEDLINE (OVID) search strategy

Database and Platform to be searched: Medline (Ovid MEDLINE® Epub Ahead of Print, In-Process & Other Non-Indexed Citations, Ovid MEDLINE® Daily and Ovid MEDLINE®) 1946 to present.

1. Colonic Neoplasms/bl [Blood]

2. Colonic Neoplasms/di [Diagnosis]

3. Colonic Neoplasms/ep [Epidemiology]

4. Colorectal Neoplasms/di [Diagnosis]

5. Colorectal Neoplasms/ep [Epidemiology]

6. Colorectal Neoplasms/bl [Blood]

7. Rectal Neoplasms/bl [Blood]

8. Rectal Neoplasms/di [Diagnosis]

9. Rectal Neoplasms/ep [Epidemiology]

10. Adenomatous Polyposis Coli/

11. Sigmoid Neoplasms/

12. Colorectal Neoplasms, Hereditary Nonpolyposis/

13. ((colorectal or bowel or colon or colonic or rectal or rectum) adj3 (cancer$ or carcinoma$ or adenoma$ or neoplas$ or metasta$ or carcinogen$ or tumour$ or tumor$ or malignan$)).ti,ab,kw.

14. or/1-13

15. exp Blood Cell Count/

16. exp Hemoglobins/

17. Blood Platelets/

18. Neutrophils/

19. Basophils/

20. Eosinophils/

21. Lymphocytes/

22. Monocytes/

23. Occult Blood/

24. Thrombocytosis/

25. Leukocytosis/

For peer review only

26. Lymphocytosis/

27. Eosinophilia/

28. Anemia/

29. Leukopenia/

30. Neutropenia/

31. Lymphopenia/

32. Thrombocytopenia/

33. Polycythemia/

34. Erythrocytes/

35. Leukocytes/

36. Pancytopenia/

37. ((blood or platelet) adj2 count$).ti,ab,kw.

38. (CBC or FBC).ti,ab,kw.

39. (blood adj2 exam$).ti,ab,kw.

40. (haematolog$ or hematolog$ or haemoglobin or hemoglobin or haematocrit or hematocrit).ti,ab,kw.

41. ((red or white) adj1 blood adj1 cell$).ti,ab,kw.

42. (mean adj1 (platelet or corpuscular) adj1 volume$).ti,ab,kw.

43. (mean adj1 corpuscular adj1 (haemoglobin or hemoglobin)).ti,ab,kw.

44. (platelet$ or basophil or basophils or eosinophil or eosinophils or lymphocyte$ or monocyte$ or neutrophil or neutrophils or erythrocyte$ or leukocyte$).ti,ab,kw.

45. (blood adj1 (test$ or draw$)).ti,ab,kw.

46. (neutrophilia or monocytosis or basophilia or anemia or anaemia or monocytopenia or eosinopenia or basopenia or thrombocytopenia or leucocytosis or lymphocytosis or eosinophilia or leucopenia or neutropenia or lymphopenia or pancytopenia or polycythemia or bicytopenia).ti,ab,kw.

47. or/15-46

48. (abnormalit$ or diagnos$ or "pre-diagnos$" or prediagnos$ or change$ or detect$ or elevat$ or distribut$ or deficien$ or identif$ or presence or indicati$ or determin$ or undiagnosed or definition$ or alteration$).ti,ab,kw.

49. (predict$ or prognos$).ti,ab,kw.

50. (risk adj1 (predict$ or marker$ or scor$)).ti,ab,kw.

51. Predictive Value of Tests/

For peer review only

52. Probability/

53. Prognosis/

54. Risk Factors/

55. Risk Assessment/

56. Incidence/

57. or/48-56

58. 14 and 47 and 57

For peer review only

PRISMA-P Checklist

Section/topic	#	Checklist item	Information reported Yes	Information reported No	Line number(s)
**ADMINISTRATIVE INFORMATION**					
**Title**					
Identification	1a	Identify the report as a protocol of a systematic review	☒	☐	2-3
Update	1b	If the protocol is for an update of a previous systematic review, identify as such	☐	☒	NA
**Registration**	2	If registered, provide the name of the registry (e.g., PROSPERO) and registration number in the Abstract	☒	☐	53; 162-163
**Authors**					
Contact	3a	Provide name, institutional affiliation, and e-mail address of all protocol authors; provide physical mailing address of corresponding author	☒	☐	6-23
Contributions	3b	Describe contributions of protocol authors and identify the guarantor of the review	☒	☐	311-316
**Amendments**	4	If the protocol represents an amendment of a previously completed or published protocol, identify as such and list changes; otherwise, state plan for documenting important protocol amendments	☐	☒	NA
**Support**					
Sources	5a	Indicate sources of financial or other support for the review	☒	☐	304-305
Sponsor	5b	Provide name for the review funder and/or sponsor	☒	☐	304-305
Role of sponsor/funder	5c	Describe roles of funder(s), sponsor(s), and/or institution(s), if any, in developing the protocol	☒	☐	305
**INTRODUCTION**					
**Rationale**	6	Describe the rationale for the review in the context of what is already known	☒	☐	67-135
**Objectives**	7	Provide an explicit statement of the question(s) the review will address with reference to participants, interventions, comparators, and outcomes (PICO)	☒	☐	138-157
**METHODS**					
**Eligibility criteria**	8	Specify the study characteristics (e.g., PICO, study design, setting, time frame) and report characteristics (e.g., years considered, language, publication status) to be used as criteria for eligibility for the review	☒	☐	194-206
**Information sources**	9	Describe all intended information sources (e.g., electronic databases, contact with study authors, trial registers, or other grey literature sources) with planned dates of coverage	☒	☐	172-181; 242-243
**Search strategy**	10	Present draft of search strategy to be used for at least one electronic database, including planned limits, such that it could be repeated	☒	☐	181

For peer review only

Section/topic	#	Checklist item	Information reported Yes	No	Line number(s)
STUDY RECORDS					
Data management	11a	Describe the mechanism(s) that will be used to manage records and data throughout the review	☒	☐	185-191; 210-213
Selection process	11b	State the process that will be used for selecting studies (e.g., two independent reviewers) through each phase of the review (i.e., screening, eligibility, and inclusion in meta-analysis)	☒	☐	185-191; 210-213
Data collection process	11c	Describe planned method of extracting data from reports (e.g., piloting forms, done independently, in duplicate), any processes for obtaining and confirming data from investigators	☒	☐	210-213; 242-243
**Data items**	12	List and define all variables for which data will be sought (e.g., PICO items, funding sources), any pre-planned data assumptions and simplifications	☒	☐	216-238
**Outcomes and prioritization**	13	List and define all outcomes for which data will be sought, including prioritization of main and additional outcomes, with rationale	☒	☐	139-158
**Risk of bias in individual studies**	14	Describe anticipated methods for assessing risk of bias of individual studies, including whether this will be done at the outcome or study level, or both; state how this information will be used in data synthesis	☒	☐	271-275
DATA					
**Synthesis**	15a	Describe criteria under which study data will be quantitatively synthesized	☒	☐	246-257
	15b	If data are appropriate for quantitative synthesis, describe planned summary measures, methods of handling data, and methods of combining data from studies, including any planned exploration of consistency (e.g., I^2, Kendall's tau)	☒	☐	251-257
	15c	Describe any proposed additional analyses (e.g., sensitivity or subgroup analyses, meta-regression)	☒	☐	260-268; 275
	15d	If quantitative synthesis is not appropriate, describe the type of summary planned	☒	☐	246-257
**Meta-bias(es)**	16	Specify any planned assessment of meta-bias(es) (e.g., publication bias across studies, selective reporting within studies)	☒	☐	271-276
**Confidence in cumulative evidence**	17	Describe how the strength of the body of evidence will be assessed (e.g., GRADE)	☐	☒	NA

For peer review only

Components of the full blood count as risk factors for colorectal cancer detection: a systematic review protocol

Journal:	*BMJ Open*
Manuscript ID	bmjopen-2019-032759.R1
Article Type:	Protocol
Date Submitted by the Author:	08-Nov-2019

Complete List of Authors:	Virdee, Pradeep; University of Oxford, Centre for Statistics in Medicine<br>Kirtley, Shona; University of Oxford, Centre for Statistics in Medicine<br>Elhussein, Leena; University of Oxford, Centre for Statistics in Medicine<br>Watkinson, Peter; Oxford University Hospitals NHS Trust, Kadoorie Centre for Critical Care research and Education<br>Holt, Tim; Oxford University, Department of Primary Care Health Sciences<br>Birks, Jacqueline; University of Oxford, Centre for Statistics in Medicine

**Primary Subject Heading**:	Oncology
Secondary Subject Heading:	Haematology (incl blood transfusion)
Keywords:	Colorectal cancer, Full blood count, Complete blood count, Early detection, Risk factor

SCHOLARONE™ Manuscripts

Title

Components of the full blood count as risk factors for colorectal cancer detection: a systematic review protocol

Authors and affiliations

Pradeep S. Virdee[1], Shona Kirtley[1], Leena Elhussein[1], Peter Watkinson[2], Tim Holt[3], Jacqueline Birks[1]

[1]Centre for Statistics in Medicine, NDORMS, University of Oxford, UK

[2]Nuffield Department of Clinical Neurosciences, John Radcliffe Hospital, Oxford, UK

[3]Nuffield Department of Primary Care Health Sciences, University of Oxford, University of Oxford, UK

Pradeep S. Virdee (PSV):	pradeep.virdee@csm.ox.ac.uk
Shona Kirtley (SK):	shona.kirtley@csm.ox.ac.uk
Leena Elhussein (LE):	leena.elhussein@csm.ox.ac.uk
Peter Watkinson (PW):	peter.watkinson@ndcn.ox.ac.uk
Tim Holt (TH):	tim.holt@phc.ox.ac.uk
Jacqueline Birks (JB):	jacqueline.birks@csm.ox.ac.uk

Corresponding author

Author: Pradeep S. Virdee

Address: CSM Botnar, Nuffield Orthopaedic Centre, Windmill Road, University of Oxford, OX3 7LD, UK

Email: pradeep.virdee@csm.ox.ac.uk

Telephone: 01865737950

For peer review only

For peer review only - http://bmjopen.bmj.com/site/about/guidelines.xhtml

Abstract

Introduction

Colorectal cancer is the fourth most common type of cancer and second most common cause of cancer-related death in the UK. The full blood count (FBC) is a blood test that may play a role in early detection of the disease. Previous studies have aimed to identify how levels of individual components, such as haemoglobin, can be used to assist diagnosis. We aim to systematically review studies to identify whether components of the FBC are risk factors for diagnosis of colorectal cancer, critically appraise the methods used to assess the association, and assess performance of components.

Methods and analysis

The MEDLINE (via OVID), EMBASE (via OVID), CINAHL (via EBSCOhost), and Web of Science databases will be searched to identify in-human studies reporting the association between levels of at least one FBC component and risk of a future diagnosis of colorectal cancer. Clincialtrials.gov and the World Health Organisation (WHO) registry will be searched to identify relevant on-going research. Search terms will include relevant Medical Subject Headings (MeSH) and Emtree headings and free-text terms relating to the FBC, colorectal cancer, and diagnosis. No date or language restrictions will be applied. Two reviewers will independently identify studies for inclusion and perform data extraction. Time intervals between the blood tests and diagnosis will form subgroups for analysis.

Ethics and dissemination

There is no direct patient involvement and only published articles will be reviewed; no ethical approval is required. Results from this review will set a foundation for intended future work on developing a new risk score for early detection of colorectal cancer, derived using FBC data. This systematic review will also provide guidance on the analysis of time to diagnosis. The model will be freely available to UK primary care practices.

For peer review only

Systematic review registration

International prospective register of systematic reviews (PROSPERO: CRD42019134400)

Strengths and limitations of this study

- The first systematic review to identify the role of components of the full blood count from a blood test in detection of colorectal cancer.
- As the number of studies reporting on the association between components of the full blood count and diagnosis of colorectal cancer is increasing over time, this review is timely.
- This systematic review will make recommendations for the development of intendend future risk scores for early detection of colorectal cancer, derived using full blood count data.
- This review will be limited to examining published articles, so the use of blood count values in diagnosis of colorectal cancer as per local practices' policy will not be included, unless published.

Introduction

Colorectal cancer is the fourth most common type of cancer in the UK, with around 41,800 new cases diagnosed in 2015[1]. It is the second most common cause of cancer-related death in the UK, with around 16,400 deaths in 2016[2]. Colorectal cancer develops slowly from pre-cancerous polyps that may be present for years before becoming malignant. It often goes unnoticed until patients start showing symptoms, such as abdominal pain, weight loss, and change in bowel habits. At this point, the cancer has usually developed to a stage where it is difficult to treat and cannot be surgically removed[3]. The stage at diagnosis heavily influences survival. Five-year survival is 95% if the cancer is diagnosed at Stage I, where the cancer is confined to the bowel lining, but 7% if at Stage IV, where the cancer has spread to other organs[4].

For peer review only

For peer review only - http://bmjopen.bmj.com/site/about/guidelines.xhtml

Patients with colorectal cancer respond well to existing interventions, such as surgery, chemotherapy, and radiotherapy, if the cancer is diagnosed at an early stage. Currently, over 50% of patients are diagnosed with late-stage cancer (Stage III and IV), with approximately half of these having metastases at diagnosis, compared with the earlier stages (Stage I and II). Their outcomes are much poorer than those diagnosed with cancer in an earlier stage, including survival[5]. Symptom-based approaches to identify risk of colorectal cancer exist, such as QCancer Colorectal, a statistical prediction model widely used in UK primary care practices[6]. However, approaches to detect cancer earlier, before overt symptoms appear, would be of considerable benefit. Early detection and removal of polyps can prevent colorectal cancer from developing and improve survival.

A full blood count (FBC) is a common blood test ordered by a doctor in both primary and secondary care, because abnormalities could relate to a wide range of diseases and conditions used in clinical practices. A FBC incudes up to 20 blood components (red blood cells, white blood cells, mean platelet volume, haemoglobin, haematocrit, mean corpuscular volume, mean corpuscular haemoglobin, mean corpuscular haemoglobin concentration, red blood cell distribution width, platelet, basophil #, basophil %, eosinophil #, eosinophil %, lymphocyte #, lymphocyte %, monocyte #, monocyte %, neutrophil #, neutrophil %).

It is known that the FBC may play a role in the diagnosis of colorectal cancer, with subtle changes in the FBC developing when the cancer is at a relatively early stage. Some studies have explored the relationship between levels of specific components of the FBC and colorectal cancer diagnosis[7, 8]. For example, anaemia in FBC data from UK primary care predicts risk of colorectal cancer and iron deficiency is an independent risk factor[8]. In the last few years, a number of individual studies have reported on the association between components of the FBC, including haemoglobin, platelet count, and red cell distribution width, and diagnosis of colorectal cancer[9-13]. Furthermore, risk scores have recently been developed using methods that incorporate FBC data as predictors for colorectal cancer

For peer review only - http://bmjopen.bmj.com/site/about/guidelines.xhtml

diagnosis. This includes the statistical prediction model QCancer Colorectal[6], which includes haemoglobin level as a predictor for risk of diagnosis, and other algorithms, such as the ColonFlag, an Israeli risk score derived recently from FBCs using machine-learning methods[14].

The aim of this review is to identify components of the FBC as potential risk factors for colorectal cancer diagnosis, given the increasing interest in the use of a FBC for early detection, and to inform the development and validation of future risk scores for early detection of colorectal cancer.

Existing systematic reviews

Systematic reviews of colorectal cancer detection already exist, four of which have been identified as reviews of blood-based characteristics. However, none of these reviews focused on the FBC blood test.

Bhardwaj *et al*[15] and Shah *et al*[16] focused on reviewing parts of the blood, such as blood-based proteins, DNA biomarkers, and gene expressions, that are not part of a FBC. Nikolou *et al*[17] reviewed blood markers that were divided into four groups: nucleic acids, cytokines, antibodies, and proteins, which are different components of the blood to the FBC. Del Giudice *et al*[18] reviewed clinical features of suspected characteristics, but this consisted primarily of symptoms reported in clinic. Their findings include the predictive performance of haemoglobin, but they do not report on the remaining blood components that form the FBC, including up to 19 other components, which are also our main interest here. Usher-Smith *et al*[19] performed a comprehensive appraisal of prediction models for risk of colorectal cancer. However, these were not necessarily models containing components of the FBC as risk factors. Additionally, other types of studies were excluded from their review, such as retrospective cohort and case-control studies, which explored the association between components of the FBC and risk of colorectal cancer diagnosis.

For peer review only - http://bmjopen.bmj.com/site/about/guidelines.xhtml

Other reviews for colorectal cancer detection have a different focus to the review described here. These include Astin *et al*[20], who reviewed the diagnostic value of symptoms, Williams *et al*[21], focusing solely on prediction models for patients who reported symptoms, and Vega *et al*[22], who summarised the available evidence concerning the diagnostic process, its pitfalls, and opportunities.

To our knowledge, there are no existing reviews identifying components of the FBC as risk factors for colorectal cancer diagnosis.

For peer review only

Research aims

In this review, we aim to identify and appraise studies that explored the association between levels of blood components from a FBC and risk of diagnosis of colorectal cancer. We aim to:

1) Identify which specific blood component(s) was investigated and whether it was considered associated with risk of diagnosis of colorectal cancer.
2) Describe the study design and methods used to assess the association. For example, whether statistical methods were used, whether blood levels were modelled as categorical or continuous, how missing data were dealt with, and the timeframe to diagnosis.
3) Describe the study population and setting. For example, whether the study used electronic health data, primary or secondary care patients, and symptomatic or asymptomatic patients.
4) Identify whether the values of the components of a FBC differ between those diagnosed with colorectal cancer and those not diagnosed.

Studies reporting the development, validation or testing of a risk score that uses FBC component(s) for colorectal cancer diagnosis, such as a prediction model, will also be included. For these studies, the following information will be collected, in addition to the points above:

5) Report the weight the blood component(s) has on risk of diagnosis of colorectal cancer. For example, this could be the coefficient from a regression model.

For peer review only - http://bmjopen.bmj.com/site/about/guidelines.xhtml

6) Identify risk factors, in addition to the components of a FBC, used to derive risk scores.
7) Report which risk scores have undergone validation, whether it was internal and/or external, and how well the risk scores performed.

Methods

This systematic review protocol follows the Preferred Reporting Items for Systematic review and Meta-Analysis Protocols (PRISMA-P) guidelines[23]. This review protocol was registered with the International Prospective Register of Systematic Reviews database on 13th May 2019 (PROSPERO: CRD42019134400). Screening of studies is expected to commence in August 2019. The review will be reported in accordance with the Preferred Reporting Items for Systematic Reviews and Meta-Analyses (PRISMA) checklist[24].

Participants

All adult (aged 18 years or above) populations will be considered, with the exception of those who are studied post-diagnosis of colorectal cancer.

Search strategy

The MEDLINE (via OVID), EMBASE (via OVID), CINAHL (via EBSCOhost), and Web of Science databases will be searched to identify publications in the medical literature that report the association between components of the FBC and diagnosis of colorectal cancer. Additionally, clincialtrials.gov and the World Health Organisation (WHO) registry will be searched to identify any relevant on-going research. Search terms will include relevant Medical Subject Headings (MeSH) and EMTREE headings, and free-text search terms, which will be searched for in the title, abstract, or keyword fields. Free-text search terms will include synonyms and related variants of the blood count, such as “platelet” or “basophil”, of colorectal cancer-related terms, such as “bowel” or “rectal”, and of detection, such as “diagnosis”

For peer review only

For peer review only - http://bmjopen.bmj.com/site/about/guidelines.xhtml

or “prediction” related terms. There will be no date or language restrictions applied to the search. The proposed full search strategy for the MEDLINE database can be found in Supplementary file 1.

Study selection

Data management

The results from each of the database searches will be downloaded into Rayyan[25], a web-tool for systematic reviews, and the full results set de-duplicated and screened. Screening of each publication will be performed independently by two reviewers. Each reviewer will read titles and abstracts to identify the study sample for analysis, using pre-specified selection criteria. Disagreements between the two reviewers will be discussed until agreement is reached. In the event that no agreement is reached, a third reviewer will be consulted for adjudication. The search results and study selection process will be reported using a PRISMA flow diagram[24].

Selection criteria

We will include any in-human primary research publication reporting the association between the value of at least one of the individual components of a FBC and the risk of a future diagnosis of colorectal cancer. For studies reporting the development and/or validation of a risk score, these will only be included if the risk score was derived using at least two risk factors, with at least one being a component of the FBC.

We will exclude abstracts and conference proceedings, as they are likely to produce incomplete data for a thorough review. As the main interest is in the recorded value of a blood component from a FBC, studies not investigating the value itself will be excluded, such as studies exploring the morphology of individual blood components. Studies using a cross-sectional design will be excluded as the data reflects a ‘snapshot’ at a certain time, hence cannot predict future risk. Clinical trials will be excluded

For peer review only

For peer review only - http://bmjopen.bmj.com/site/about/guidelines.xhtml

as our interest is in FBC data, not intervention. Existing systematic reviews, correspondence, and case studies pertaining to single individuals will be excluded.

Data extraction

Data management

Data extracted from the publications included for analysis is intended to be recorded in the Research Electronic Data Capture (REDCap) electronic tool[26]. Two reviewers will read full-text publications and independently extract data from each study. Disagreements between the two reviewers will be resolved by a third reviewer.

Data items

Data extracted will include items from the PRISMA checklist[24]. Data specific to this study will be extracted using a self-developed data extraction form. This form will be piloted on a small number of publications in the final sample; the publications will be selected randomly and the number chosen for piloting will depend on number of publications included in the sample for analysis. The study aims specify the data items for extraction, which will include the following:

- Study characteristics (such as authors, year of publication, sample size)
- Study design (such as prospective, cohort, case-control)
- Study setting (such as geographical location, primary or secondary care, electronic health record data)
- Population characteristics (such as age, gender, symptomatic/asymptomatic)
- Outcome details (such as number of cases/controls, timeframe)
- Blood component details (such as which component was analysed, blood levels used, its association with risk of colorectal cancer)
- Methods applied to assess the association (such as how missing data were handled, whether blood levels were categorised, statistical methods used)

For peer review only

For peer review only - http://bmjopen.bmj.com/site/about/guidelines.xhtml

- Differences between those with and without a diagnosis of colorectal cancer, where possible (such as whether blood levels differ between the two groups).

For the subset of studies reporting the development, validation or testing of a risk score for colorectal cancer diagnosis that incorporates FBC data, the following data items will also be collected:

- Weight of blood component(s) on risk (such as model coefficient, 95% confidence interval)
- Risk factors used in addition to the blood component(s) (such as age, gender)
- Measures of discrimination and calibration (such as sensitivity, specificity)

Data analysis and synthesis

Missing data

We will contact the publication’s authors to request missing or incomplete data. If the data are not obtained, we will provide a description of the missing data for each study.

Analysis methods

Quantitative data will be summarised using descriptive statistics, such as means with standard deviations or medians with inter-quartile range for continuous data and counts with proportions for categorical data, and narrative synthesis. Summaries will also be provided in graphical and tabular form.

Where appropriate and feasible, we will use random-effects meta-analysis and forest plots to pool data across studies, with the I^2 statistic to assess heterogeneity. The application of statistical methods for analysis will be informed by the number of publications with common measures. For example, if many case-control studies report effect estimates, such as odds ratios or risk ratios of blood component levels between those with and without a diagnosis of colorectal cancer, effect estimates

For peer review only

For peer review only - http://bmjopen.bmj.com/site/about/guidelines.xhtml

will be pooled using a random-effects meta-analysis. For all statistical analyses, a two-sided 5% significance level will be used.

Subgroups for analysis

The time-period between the blood test and diagnosis of colorectal cancer will be collected, such as whether the studies report two-year or five-year risk of diagnosis. It is intended that time intervals will be separated into bands, such as 6-monthly or yearly intervals before diagnosis, forming subgroups for analysis. Analyses will be performed for each time band separately. These results will set a foundation for intended future work on the analyses of time to diagnosis.

Planned future work will use findings from this review to develop a new risk score for early detection of colorectal cancer in the UK. Hence, studies performed in the UK population will form a subgroup for analysis.

Assessment of bias

Risk of bias, such as analysis bias and publication bias, in each study will be assessed using tools appropriate to the design of the study. For example, the Quality In Prognosis Studies (QUIPS) tool[27] may be used for studies of associations and the Cochrane Prediction model Risk Of Bias ASsessment Tool (PROBAST)[28] may be used for the subset identified as prediction modelling studies. Studies considered to have a high risk of bias will be excluded in a sensitivity analysis.

Ethics and dissemination

Ethical approval is not required for this systematic review as there will be no direct patient investigations in this study and only published articles will be systematically reviewed. Results from this systematic review will be published in an open access journal.

For peer review only - http://bmjopen.bmj.com/site/about/guidelines.xhtml

Patient and public involvement

Patients and members of the public reviewed this protocol and will review the results to apply a patient perspective to the research.

Discussion

Many studies have explored the role that individual components of a FBC may have in the diagnosis of colorectal cancer. Such exploration has recently become popular. However, no changes have been incorporated widely in general practice to make use of such data for early detection of the disease. Despite existing methods for colorectal cancer detection, such as screening, and methods to assist detection, such as the widely-used prediction model QCancer Colorectal, the lack of early detection remains a major health problem, with the majority of colorectal cancers in the UK diagnosed at a late stage[5]. The ability to identify cases of colorectal cancer at a time point earlier than existing methods would be of considerable benefit and could save many lives.

Our team has available a set of FBC data from a large cohort of patients from UK primary care practices. However, optimal strategies for modelling the data, such as handling biologically implausible blood values and analysing changes over time, are unclear. Having appraised individual studies, this review will provide guidance on the specific components to analyse, i.e. those identified as risk factors, and appropriate statistical modelling strategies to implement. Intended future work includes developing and validating a new risk score for early detection of colorectal cancer, derived using statistical modelling of FBC components, based off the findings of this review. This model will be made freely available to primary care practices in the UK. Primary care practices will have the opportunity to use the model to identify the likelihood of an individual being diagnosed with colorectal cancer in the future.

For peer review only

For peer review only - http://bmjopen.bmj.com/site/about/guidelines.xhtml

Funding

This study is funded by the National Institute for Health Research (NIHR) Doctoral Research Fellowship programme (DRF-2018-11-ST2-057). The funders had no role in the development of this protocol.

Competing interests

The authors declare that they have no competing interests.

Authors' contributions

PSV, TH, and JB developed the study and research question. PSV developed this systematic review protocol. SK developed the search strategy, with input from PSV and LE and oversight from PW, TH, and JB. PSV developed the data extraction sheet, with input from LE and oversight from PW, TH, and JB. PSV and SK will perform the final study search. PSV and LE will perform screening, data extraction, and analysis under the supervision of PW, TH, and JB. PSV drafted this protocol and all authors developed and approved the final manuscript before submission.

Acknowledgements

The authors would like to thank Pete Wheatstone, Margaret Ogden, and Julian Ashton for their input into the protocol as patient and public involvement (PPI) representatives.

References

[1] Cancer Research UK. Bowel Cancer Incidence. https://www.cancerresearchuk.org/health-professional/cancer-statistics/statistics-by-cancer-type/bowel-cancer/incidence#heading-Two (last accessed 3 May 2019).

For peer review only - http://bmjopen.bmj.com/site/about/guidelines.xhtml

[2] Cancer Research UK. Bowel Cancer Mortality. https://www.cancerresearchuk.org/health-professional/cancer-statistics/statistics-by-cancer-type/bowel-cancer/mortality (last accessed 3 May 2019).

[3] Winawer S.J., Zauber A.G., Ho M.N., O'Brien M.J., *et al* (1993). Prevention of Colorectal Cancer by Colonoscopic Polypectomy. *N. Engl. J. Med*. 329:1977–1981.

[4] Cancer Research UK. Bowel Cancer Survival Statistics. Available at https://www.cancerresearchuk.org/health-professional/cancer-statistics/statistics-by-cancer-type/bowel-cancer/survival#heading-Three (last accessed 3 May 2019).

[5] Cancer Research UK. Bowel Cancer Incidence. Available at http://www.cancerresearchuk.org/health-professional/cancer-statistics/statistics-by-cancer-type/bowel-cancer/incidence#heading-Three (last accessed 3 May 2019).

[6] Hippisley-Cox J., Coupland C. (2015). Development and validation of risk prediction algorithms to estimate future risk of common cancers in men and women: prospective cohort study. *BMJ Open.* 5:e007825.

[7] Spell D.W., Jones D.V. Jr, Harper W.F., Bessman D.J., *et al* (2004). The value of a complete blood count in predicting cancer of the colon. *Cancer Detection and Prevention*. 28:37–42.

[8] Hamilton W., Lancashire R., Sharp D., Peters T.J., *et al* (2008). The importance of anaemia in diagnosing colorectal cancer: a case-control study using electronic primary care records. *Br. J. Cancer*. 98:323–327.

For peer review only

[9] Bailey SER., Ukoumunne O.C., Shephard E.A., Hamilton W., *et al* (2017). Clinical relevance of thrombocytosis in primary care: a prospective cohort study of cancer incidence using English electronic medical records and cancer registry data. *Br. J. of Gen Pract*. 67(662):e405-e413.

[10] Goshen R., Mizrahi B., Akiva P., Kinar Y., *et al* (2017). Predicting the presence of colon cancer in members of a health maintenance organisation by evaluating analytes from standard laboratory records. *British Journal of Cancer*. 116:944–950.

[11] Pilling L.C., Atkins J.L., Kuchel G.A., Ferrucci L., *et al* (2018). Red cell distribution width and common disease onsets in 240,477 healthy volunteers followed for up to 9 years. *PLOS ONE*. 13(9):e0203504.

[12] Song Y., Huang Z., Kang Y., Lin Z., *et al* (2018). Clinical Usefulness and Prognostic Value of Red Cell Distribution Width in Colorectal Cancer. *BioMed Research International*. 9858943:1-7.

[13] Ankus E., Price S.J., Ukoumunne O.C., Hamilton W., *et al* (2018). Cancer incidence in patients with a high normal platelet count: a cohort study using primary care data. *Family Practice*. 35(6):671–675.

[14] Kinar Y., Kalkstein N., Akiva P., Levin B., *et al* (2016). Development and validation of a predictive model for detection of colorectal cancer in primary care by analysis of complete blood counts: a binational retrospective study. *J Am Med Inform Assoc*. 23:879–890.

[15] Bhardwaj M., Gies A., Werner S., Schrotz-King P., *et al* (2017). Blood-Based Protein Signatures for Early Detection of Colorectal Cancer: A Systematic Review. *Clinical and Translational Gastroenterology*. 8:e128.

For peer review only - http://bmjopen.bmj.com/site/about/guidelines.xhtml

[16] Shah R., Jones E., Vidart V., Kuppen P.J., *et al* (2014). Biomarkers for Early Detection of Colorectal Cancer and Polyps: Systematic Review. *Cancer Epidemiol Biomarkers Prev*. 23(9):1712-1728.

[17] Nikolaou S., Qiu S., Fiorentino F., Rasheed S., *et al* (2018). Systematic review of blood diagnostic markers in colorectal cancer. *Techniques in Coloproctology*. 22:481–498.

[18] Del Giudice M.E., Vella E.T., Hey A., Simunovic M., *et al* (2014). Systematic review of clinical features of suspected colorectal cancer in primary care. *Canadian Family Physician*. 60:e405-e415.

[19] Usher-Smith J.A., Walter F.M., Emery J.D., Win A.K., *et al* (2016). Risk Prediction Models for Colorectal Cancer: A Systematic Review. *Cancer Prev Res*. 9(1):13–26.

[20] Astin M., Griffin T., Neal R.D., Rose P., *et al* (2011). The diagnostic value of symptoms for colorectal cancer in primary care: a systematic review. *Br. J. of Gen Pract*. 61(586):e231-e243.

[21] Williams T.G.S., Cubiella J., Griffin S.J., Walter F.M., *et al* (2016). Risk prediction models for colorectal cancer in people with symptoms: a systematic review. *BMC Gastroenterology*. 16(63):1-16.

[22] Vega P., Valentín F., Cubiella J. (2015). Colorectal cancer diagnosis: pitfalls and opportunities. *World J Gastrointest Oncol*. 7(12):422-433.

[23] Moher D., Shamseer L., Clarke M., Ghersi D., *et al* (2015). Preferred Reporting Items for Systematic Review and Meta-Analysis Protocols (PRISMA-P) 2015 statement. *Systematic Reviews*. 4(1):1.

[24] Moher D., Liberati A., Tetzlaff J., Altman D.G., *et al* (2009). Preferred Reporting Items for Systematic Reviews and Meta-Analyses: The PRISMA Statement. *PLoS Med*. 6(7):e1000097.

For peer review only

For peer review only - http://bmjopen.bmj.com/site/about/guidelines.xhtml

[25] Mourad O., Hammady H., Fedorowicz Z., Elmagarmid A. (2016). Rayyan — a web and mobile app for systematic reviews. *Systematic Reviews*. 5:210.

[26] Harris P.A., Taylor R., Thielke R., Payne J., *et al* (2009). Research electronic data capture (REDCap) – A metadata-driven methodology and workflow process for providing translational research informatics support. *J Biomed Inform*. 42(2):377-81.

[27] Hayden J.A., van der Windt D.A., Cartwright J.L., Cote P.C. (2013). Assessing Bias in Studies of Prognostic Factors. *Annals of Internal Medicine*. 158:280-286.

[28] Wolff R.F., Moons K.G.M., Riley R.D., Whiting P.F., *et al* (2019). PROBAST: A Tool to Assess the Risk of Bias and Applicability of Prediction Model Studies. *Annals of Internal Medicine*. 170:51-58.

For peer review only

For peer review only - http://bmjopen.bmj.com/site/about/guidelines.xhtml

Proposed MEDLINE (OVID) search strategy

Database and Platform to be searched: Medline (Ovid MEDLINE® Epub Ahead of Print, In-Process & Other Non-Indexed Citations, Ovid MEDLINE® Daily and Ovid MEDLINE®) 1946 to present.

1. Colonic Neoplasms/bl [Blood]
2. Colonic Neoplasms/di [Diagnosis]
3. Colonic Neoplasms/ep [Epidemiology]
4. Colorectal Neoplasms/di [Diagnosis]
5. Colorectal Neoplasms/ep [Epidemiology]
6. Colorectal Neoplasms/bl [Blood]
7. Rectal Neoplasms/bl [Blood]
8. Rectal Neoplasms/di [Diagnosis]
9. Rectal Neoplasms/ep [Epidemiology]
10. Adenomatous Polyposis Coli/
11. Sigmoid Neoplasms/
12. Colorectal Neoplasms, Hereditary Nonpolyposis/
13. ((colorectal or bowel or colon or colonic or rectal or rectum) adj3 (cancer$ or carcinoma$ or adenoma$ or neoplas$ or metasta$ or carcinogen$ or tumour$ or tumor$ or malignan$)).ti,ab,kw.
14. or/1-13
15. exp Blood Cell Count/
16. exp Hemoglobins/
17. Blood Platelets/
18. Neutrophils/
19. Basophils/
20. Eosinophils/
21. Lymphocytes/
22. Monocytes/
23. Occult Blood/
24. Thrombocytosis/
25. Leukocytosis/

For peer review only

26. Lymphocytosis/

27. Eosinophilia/

28. Anemia/

29. Leukopenia/

30. Neutropenia/

31. Lymphopenia/

32. Thrombocytopenia/

33. Polycythemia/

34. Erythrocytes/

35. Leukocytes/

36. Pancytopenia/

37. ((blood or platelet) adj2 count$).ti,ab,kw.

38. (CBC or FBC).ti,ab,kw.

39. (blood adj2 exam$).ti,ab,kw.

40. (haematolog$ or hematolog$ or haemoglobin or hemoglobin or haematocrit or hematocrit).ti,ab,kw.

41. ((red or white) adj1 blood adj1 cell$).ti,ab,kw.

42. (mean adj1 (platelet or corpuscular) adj1 volume$).ti,ab,kw.

43. (mean adj1 corpuscular adj1 (haemoglobin or hemoglobin)).ti,ab,kw.

44. (platelet$ or basophil or basophils or eosinophil or eosinophils or lymphocyte$ or monocyte$ or neutrophil or neutrophils or erythrocyte$ or leukocyte$).ti,ab,kw.

45. (blood adj1 (test$ or draw$)).ti,ab,kw.

46. (neutrophilia or monocytosis or basophilia or anemia or anaemia or monocytopenia or eosinopenia or basopenia or thrombocytopenia or leucocytosis or lymphocytosis or eosinophilia or leucopenia or neutropenia or lymphopenia or pancytopenia or polycythemia or bicytopenia).ti,ab,kw.

47. or/15-46

48. (abnormalit$ or diagnos$ or "pre-diagnos$" or prediagnos$ or change$ or detect$ or elevat$ or distribut$ or deficien$ or identif$ or presence or indicati$ or determin$ or undiagnosed or definition$ or alteration$).ti,ab,kw.

49. (predict$ or prognos$).ti,ab,kw.

50. (risk adj1 (predict$ or marker$ or scor$)).ti,ab,kw.

51. Predictive Value of Tests/

For peer review only

52. Probability/

53. Prognosis/

54. Risk Factors/

55. Risk Assessment/

56. Incidence/

57. or/48-56

58. 14 and 47 and 57

For peer review only

PRISMA-P Checklist

Section/topic	#	Checklist item	Information reported: Yes	Information reported: No	Line number(s)
**ADMINISTRATIVE INFORMATION**					
**Title**					
Identification	1a	Identify the report as a protocol of a systematic review	☒	☐	2-3
Update	1b	If the protocol is for an update of a previous systematic review, identify as such	☐	☒	NA
**Registration**	2	If registered, provide the name of the registry (e.g., PROSPERO) and registration number in the Abstract	☒	☐	53; 162-163
**Authors**					
Contact	3a	Provide name, institutional affiliation, and e-mail address of all protocol authors; provide physical mailing address of corresponding author	☒	☐	6-23
Contributions	3b	Describe contributions of protocol authors and identify the guarantor of the review	☒	☐	311-316
**Amendments**	4	If the protocol represents an amendment of a previously completed or published protocol, identify as such and list changes; otherwise, state plan for documenting important protocol amendments	☐	☒	NA
**Support**					
Sources	5a	Indicate sources of financial or other support for the review	☒	☐	304-305
Sponsor	5b	Provide name for the review funder and/or sponsor	☒	☐	304-305
Role of sponsor/funder	5c	Describe roles of funder(s), sponsor(s), and/or institution(s), if any, in developing the protocol	☒	☐	305
**INTRODUCTION**					
**Rationale**	6	Describe the rationale for the review in the context of what is already known	☒	☐	67-135
**Objectives**	7	Provide an explicit statement of the question(s) the review will address with reference to participants, interventions, comparators, and outcomes (PICO)	☒	☐	138-157
**METHODS**					
**Eligibility criteria**	8	Specify the study characteristics (e.g., PICO, study design, setting, time frame) and report characteristics (e.g., years considered, language, publication status) to be used as criteria for eligibility for the review	☒	☐	194-206

For peer review only

Section/topic	#	Checklist item	Information reported Yes	Information reported No	Line number(s)
**Information sources**	9	Describe all intended information sources (e.g., electronic databases, contact with study authors, trial registers, or other grey literature sources) with planned dates of coverage	☒	☐	172-181; 242-243
**Search strategy**	10	Present draft of search strategy to be used for at least one electronic database, including planned limits, such that it could be repeated	☒	☐	181
STUDY RECORDS					
Data management	11a	Describe the mechanism(s) that will be used to manage records and data throughout the review	☒	☐	185-191; 210-213
Selection process	11b	State the process that will be used for selecting studies (e.g., two independent reviewers) through each phase of the review (i.e., screening, eligibility, and inclusion in meta-analysis)	☒	☐	185-191; 210-213
Data collection process	11c	Describe planned method of extracting data from reports (e.g., piloting forms, done independently, in duplicate), any processes for obtaining and confirming data from investigators	☒	☐	210-213; 242-243
**Data items**	12	List and define all variables for which data will be sought (e.g., PICO items, funding sources), any pre-planned data assumptions and simplifications	☒	☐	216-238
**Outcomes and prioritization**	13	List and define all outcomes for which data will be sought, including prioritization of main and additional outcomes, with rationale	☒	☐	139-158
**Risk of bias in individual studies**	14	Describe anticipated methods for assessing risk of bias of individual studies, including whether this will be done at the outcome or study level, or both; state how this information will be used in data synthesis	☒	☐	271-275
DATA					
**Synthesis**	15a	Describe criteria under which study data will be quantitatively synthesized	☒	☐	246-257
	15b	If data are appropriate for quantitative synthesis, describe planned summary measures, methods of handling data, and methods of combining data from studies, including any planned exploration of consistency (e.g., I^2, Kendall's tau)	☒	☐	251-257
	15c	Describe any proposed additional analyses (e.g., sensitivity or subgroup analyses, meta-regression)	☒	☐	260-268; 275
	15d	If quantitative synthesis is not appropriate, describe the type of summary planned	☒	☐	246-257
**Meta-bias(es)**	16	Specify any planned assessment of meta-bias(es) (e.g., publication bias across studies, selective reporting within studies)	☒	☐	271-276
**Confidence in cumulative evidence**	17	Describe how the strength of the body of evidence will be assessed (e.g., GRADE)	☐	☒	NA

For peer review only
